# What isn’t public health?

**DOI:** 10.1057/s41271-023-00404-x

**Published:** 2023-04-03

**Authors:** Samuel Brookfield

**Affiliations:** School of Public Health, The University of Queensland, Brisbane, Australia

**Keywords:** Values, Agency, Politics, Public policy, Public health

## Abstract

By recognizing the structural causes of health and illness, public health has often been associated with values of compassion and solidarity, and a relational understanding of human agency. Rather than supporting the consistent integration and application of these insights, however, public health is now sometimes invoked more as a rhetorical move, used to construct issues as simple questions of neoliberal scientistic rationalism. Public health practitioners must reckon, therefore, with how the field can be discursively deployed in the public square, for multiple divergent political ends. If public health is always positioned as a value-neutral and detached scientific approach to addressing complex subjects, from drug use to pandemics, it not only fails to connect with the arguments of its critics, but further divorces what was once called the public health ‘movement’ from the strong and progressive political and theoretical positions it was founded upon and should advocate for today.

## Key messages

- It is necessary to critique the way public health can be constructed as value neutral, and thereby used for different political ends whilst also being limited in its impact on complex issues.
- A public health approach entails certain inherent theoretical and political commitments, including perspectives on human agency, a relational form of ethics, and values such as solidarity and compassion.
- Public health should be understood and invoked more as a particular analytical stance, with an explicit value system, rather than a circumscribed area of detached scientific inquiry.

## Introduction

The rhetoric of constructing particular problems as ‘public health’ issues has ebbed and flowed around topics including obesity [1], harmful drug use [2], sexual harassment [3], and domestic violence [4, 5], to gun violence [6], police brutality [7], racism [8], and war [9]. Often public health is positioned discursively as a more neutral and scientific way of understanding these various complex health-related phenomena. As part of its meteoric rise in profile during the COVID-19 pandemic, many invoked public health as an antidote to the division, confusion, and general melee that engulfed most responses to the crisis. Appeals to ‘follow the science’, however, struggled to gain credibility and public consensus, in a dynamic environment where the political, ethical, and cultural aspects of pandemic response were often under-managed and under-addressed [10, 11]. Public health language and rhetoric can function, when employed uncritically, as a normative driver, able to implicitly and falsely separate a particular subject from other complex issues like ethics, market regulation, politics, or religious belief. If the theoretical and political assumptions inherent to public health research and policy go unacknowledged, or are even denied by practitioners, then this confusion will continue, and the health of the public will suffer.

There has been lively and ongoing debate among scholars regarding the ‘boundary problem’ of public health. This problem refers to the lack of consensus about what should be considered the rightful purview of public health practitioners [12–14]. A frequent lack of reference to the boundary problem in public and political discourse presents a secondary challenge. Public health has internal theoretical issues, but there is an additional class of issue presented when those in public health assert that it is a clearly defined and value-neutral approach. Arguing that merely ‘following the science’ will lead the way out of challenges like pandemic management is an example of the naturalistic fallacy: there is nothing inherent to any assessment of risks, costs, and benefits that will tell policy makers what to do, without an explicit value structure and political theory in which to situate it [15].

If public health is employed only as an uncomplicated and detached scientific approach to addressing complex subjects, from drug use to pandemics, then it not only fails to connect with the arguments of its critics, but divorces what was once called the public health ‘movement’ [16] from the strong and progressive political and theoretical positions upon which it was founded [17]. In this Viewpoint, therefore, I critique the way public health can be constructed as value neutral and used for different political ends while also being limited in its impact on complex issues; explore the theoretical and political commitments inherent in a public health approach, including perspectives on human agency, a relational form of ethics, and values such as solidarity and compassion; and argue for an understanding of public health as a particular analytical stance, with an explicit value system, rather than a circumscribed area of detached scientific inquiry.

## Defining public health

Defining the scope of public health remains a consequentially elusive goal. Responses range from Rudolf Virchow’s [18] claim that politics is simply ‘medicine at a grand scale’, to a narrower pursuit of contemporary ‘surrogate values’ in the form of various health outcomes that enable policymakers to prioritize and pursue specific interventions [19]. The Public Health Association Australia [20] acknowledges that their field “encompasses nearly every aspect of our lives and surrounding environment making it difficult to find a definition broad enough to illustrate its complexity”. The World Health Organisation (WHO) paints with a similarly broad brush, defining public health as “the art and science of preventing disease, prolonging life and promoting health through the organized efforts of society” [21]*.* This definition is expansive, and already begs the question: Which of a society’s ‘organised efforts’ would not be relevant to this pursuit?

These modern definitions of public health have been winnowed down to vaguer and less value-laden language that avoids committing adherents to specific courses of action and allows application of the label of public health to politically or value-divergent projects. The foundation of public health, however, originally had a more prescriptive outlook. An older articulation by public health pioneer C.E.A. Winslow [22], from which the WHO definition is clearly derivative, illustrates this:

Public health is the science and the art of preventing disease, prolonging life and promoting physical health and efficiency through organised community efforts for the sanitation of the environment, the control of community infections, the education of the individual in principles of personal hygiene, the organisation of medical and nursing service for the early diagnosis and preventative treatment of disease, and the development of social machinery which will ensure to every individual in the community a standard of living adequate for the maintenance of health.

This definition provides an agenda for action, a series of specific interventions that follow from the original broader intentions to prolong life and promote health through collective effort. Public health is involved here in the implementation of ‘social machinery’ to achieve the humanist ends of equitable health, language which might invite fairly sharp and politicised responses if used today. This aspirational blueprint followed directly, however, from the politically informed programme of discoveries and arguments on which public health had been built.

The public health approach emerged from mid-nineteenth century developments in medicine and epidemiology that illuminated concepts like contagion, occupational disease, and the essential nature of sanitation [17]. Proponents used these insights to argue for improvements in living and working conditions for the poor, and more humane management of places such as psychiatric institutions [17, 23]. This approach tied public health to economic and cultural ideas of progress, compassion, and solidarity, in the face of exponential industrialization and yawning inequality.

Redefining health as a publicly produced, emergent phenomena was driven by figures no less political than Fredrich Engels [24], who starkly articulated the concept of ‘social murder’ to describe the means by which the untimely deaths of the working class were inflicted. This phrase has ongoing currency particularly in response to events such as the Grenfell Tower Fire [25], a disaster in which 72 people died that was connected to unsafe building materials and a wider context of austerity and corruption in the UK. Early social-medical philosophers such as Virchow similarly drew direct links from the health of the people to economic conditions, and to the need for structural and political solutions [17]. These broad thinkers founded public health on the idea that health and disease were distributed through society as a result of social problems, based on a critical awareness of class disadvantage, the ravages of hierarchical power, and the dehumanizing environments conjured by the industrial revolution. This more elaborated programme contrasts with the minimal claim that health can be promoted through the ‘organized efforts of society’, which could arguably include our increasingly neoliberal projects of biomedical research and individualized clinical care.

More than the technical invention of particular statistical methods or tools of measurement, public health entails a logic of how to understand health and the drivers of human behaviour. A public health approach can help identify the root causes of social ills, whether a specific infection outbreak like cholera, or a more dispersed enemy like institutionalized discrimination. Any definition of public health will always depend, therefore, upon a pre-existing value system and political theory telling practitioners what kinds of knowledge to seek and what problems to address. Public health did not discover that social problems were the cause of ill health; it discovered the phenomena of socially produced health problems themselves, giving us a language to describe how infectious diseases, addiction, obesity, or violence propagate in different times and places due to the systems and structures in place. The early public health campaigners were like fish coming up with a word for water. It is an insight which we have resisted ever since.

## Public health values

Prior to determining what values should guide public health in a particular setting, we must acknowledge the role values will always play is essential. There is no value-neutral ‘view from nowhere’ through which to conduct research or write policy in response to objective scientific findings [26, 27], and it is damaging to the intellectual force of public health analyses when they are presented that way. Even if we aspire to be value-neutral, all that means is that we value value-neutrality. As argued by Coggon [12]:

By treating something as public, we invite analysis from political philosophy, and necessarily any substantive response to a public health issue will imply more fundamental points about the nature, basis, and scope of political obligation.

Obscuring the value commitments of a public health approach can also blind practitioners and critics alike to cultural and political expediencies that continue to determine what public health is capable of in terms of generating and applying knowledge [28]. A paper from the Centre for Infectious Disease Research and Policy [29] at the University of Minnesota discussing COVID-19 touches on this exact point. The authors highlight that at the time of publication the political nature of decisions regarding pandemic response were becoming more acute as governments tried to navigate the ‘next step’ dilemma, after having invoked strict lockdowns for significant periods of time.

… a great deal of COVID-19 rhetoric so far has given the misimpression that the purpose of lockdown was to end the crisis. … Please note that none of them will end the crisis. This is not a scientific dilemma, even though the discussion (and the implementation!) must be informed by evolving scientific knowledge. It is a political dilemma, because it is about values. It is a choice that political leaders should make in consultation not just with experts but with the public at large.

This argument acknowledges the necessity of politics and value judgements to guide and implement scientific findings. Kenny, Sherwin, and Bayliss [30] emphasise this point in their discussion of relational ethics in the context of pandemic management, linking the practice of public health with necessary values of relational autonomy, solidarity, and social justice. They understand public health in the context of a necessarily political agenda, where it must *‘*attend to the needs of all, especially the most vulnerable and systematically disadvantaged members of society’ [30]. Although they may be widely if superficially subscribed, these values are not universal. Public health practitioners must be attuned to this wider value-shaped environment when developing and applying findings. Public health practitioners will be frustrated if they attempt to translate their analysis into meaningful action in a context where the relevant political, economic, and healthcare systems are out of sync with the implications of their research.

An expansive and politically committed public health is not without its risks. Rothstein [13] argues that public health be restricted to a narrow ‘jurisdiction’, guided by a perhaps less lofty set of values, to reduce the risk of government overreach in its pursuit of supposed common goods. Rothstein buttresses this argument by plainly admitting that structural factors that may influence health such as poverty must be considered outside of the scope of public health, thereby excluding explicit values such as equity or class solidarity. He argues that if the name public health is to have meaning, we must draw the line somewhere, and the field is strengthened by drawing it conservatively. As Coggon [12] highlights, however, drawing this boundary “may be an impossible task, a task that calls for arbitrary distinctions, or one that involves an incomplete analytic focus”. Imposing a jurisdiction on public health does not resolve the issue of how to fully analyse health without allusion to far-reaching structural factors.

It can be cogently argued that there is a meaningful distinction between *public* health and *private* welfare, both of which can have broader ‘social determinants’ [31]. But here we arrive again at the relationship between public health values and definitions of the field. We cannot pursue public health without a particular set of values regarding the social responsibility for ensuring both public health and private welfare. Nor can we exclude anything from public health without making a value judgement about where to draw the line between the two. From this perspective, public health is not necessarily a specific subject of study, from which certain things can be excluded, but a way of looking at the world. Are public health professionals and institutions the best agents for addressing some aspects of health? This is a reasonable question for debate. But rather than an empirically answerable question, the answer will shift depending on the values of the questioner.

## Public health and agency

The analytical stance of public health regarding structural influences on human behaviour also entails philosophical and political positions on personal agency that can be challenging to apply [14, 32]. On one hand the allusion to social problems impacting wellbeing has been a basic and obvious suggestion since public health’s inception. Anyone living in a nineteenth century industrialised city would have agreed that living conditions were relevant to the health of the working class. On the other hand, this was also a radical suggestion that contravened basic political and philosophical assumptions of the Enlightenment. These assumptions have only been further reified and reinforced by modern neoliberalism, the main idea of which being that each person is a discrete and autonomous individual, responsible for the rational and purposeful management of their own health [33].

An often unacknowledged implication of the public health approach is that individual agency being restrained, partial, or even absent is a normal state of affairs, given that health outcomes are never attributable only to the free decisions of an individual [32, 34, 35]. It is only when we acknowledge how structural and systemic factors influence the actions of individuals, in ways that challenge many people’s fundamental ideas of themselves as autonomous actors, that the language of public health as applied to human behaviours can make any sense. Scholars continue to hotly contest whether some agentic force completely internal to an individual person contributes to their behaviour [35–37]. If one recognizes, however, that individual autonomy is at least restricted, then the ideological space is created in which public health analysis can flourish. Limited agency can be a disruptive and unappealing idea, however, when contrasted with the personal values and beliefs of many people. This view therefore continues to be a politically unviable framework in cultures that prize self-determination.

Public health itself has had an uneasy relationship with its own insights regarding individual autonomy. Jennings [14] highlights this issue by situating public health within an individualizing liberal tradition. The assumptions of liberalism, as a political system founded on the sovereign individual, can constrain the imagination and possibilities of public health analysis. As we continue to learn more about the profound interdependence between individual health and the broader social, cultural, and environmental context, the more public health practitioners must allude to a *‘*richer moral meaning’ in justifying policy recommendations that fundamentally contradict individualism in favour of structural change [14].

A primary concern about this understanding of personal agency as limited or inconsequential is its potential for justifying coercion, paternalism, and state power. We must consider that when something becomes designated a public health issue, we are giving implicit license for politically shaped and state-based institutions to intervene. Public health language can be used to provoke government action. But we must, at least, consider the potentially invasive and ethically complex actions the state may take. This is especially important today where health is increasingly integrated with neoliberal regimes of state and self-surveillance, such as through the use of digital behaviour tracking applications to reduce drug use [38], and the expansion of paternalistic ‘surveillance medicine’ to shape lifestyle choices [39]. As Dew [39] argues in his discussion of public health as a ‘cult of humanity’, its ambivalent relationship with power structures means that “it can play a role in tempering the ‘monstrous’ tendencies of the state, but can also, at particular historical moments, play a role in facilitating the monstrous state.” To guard against the potentially monstrous effects of unexamined public health, therefore, implicit theories on issues as fundamental as human agency and what constitutes a ‘public’ concern must be made explicit and justified.

## What isn’t public health?

Rather than debating whether or not gun violence or domestic violence or harmful drug use ‘counts’ as a public health issue, it may be more instructive to ask what is not public health? Are there any aspects of health that are not available to a systemic, population level analysis? I would claim that there is not.

How about reducing a supposedly very individualized adverse health outcome such as elective cosmetic surgery complications? From a public health perspective, maybe we should look at the factors shaping the standards and forms of oversight for surgeons and other staff in the relevant clinics, and the various economic incentives that determine this framework. Also, we could assess the social and cultural factors that define the population that receive these surgeries, their general compliance with post-surgical care, and their other various strengths and vulnerabilities. There may also be broader regulatory questions regarding the ethics of how these surgeries are advertised, and how their allure is shaped by aspects of race, class, gender, and heteronormativity (see for example Alvaro [40]). Scholars such as Rothstein [13] may argue that some of these are questions for the humanities or the social sciences, but if the answers can be used to inform health policy, then there is no internal logic whereby public health can seal itself off from this wider landscape of inquiry.

Falls in aged care facilities, netball injuries, electrocutions from Christmas lights, unicycle accidents: anything can be a public health issue, because any health issue will inescapably include some component of structural and systemic issues which contribute to its incidence. Once we acknowledge the structures in which we exist, and the systems in which we are embedded, then no health outcome or process can be viewed as occurring outside of that definitionally all-encompassing structure or system. No fish can be explained without alluding to water at some point.

When addressing an issue of social concern, the question should not be whether something deserves the label of public health. We should instead be asking whether the value system ascribed to by the practitioner, group, or institution that is tackling the issue supports applying the analytical stance of public health, along with the entailed objectives, values, and theoretical assumptions described above. A problem becomes a problem of the public when we agree that it will not be solved by sovereign individuals making different and better choices based on enlightened rationality [41]. Thus, the question of whether something counts as public health often boils down to a debate over whether a behaviour should be considered a matter of personal or state responsibility. That is, anything can be viewed as a personal or public issue, depending on the analytical stance adopted.

Many people feel something like gun violence can be prevented by individuals choosing not to be violent. For them, it as an issue of ‘mental health’ for a few specific individuals, whether they be isolated, ideological mass murderers or gang members embedded in a sociocultural environment where violence is normalised. Others view gun violence as arising from social fragmentation, austerity, gun availability, and intergenerational cycles of trauma [42, 43]. Both are ‘right’ because both are talking about the same issue in an internally coherent way with different analytical frameworks, and with different theories of human agency. Neither public health nor advocates of personal responsibility can win the argument when it is structured this way. Advancing the argument that gun violence is a public health issue will always struggle if these theoretical assumptions underlying it remain unacknowledged.

Ignoring these theoretical differences may mean debates spiral on for a lifetime. For decades the United States (U.S.) government authorities barred the U.S. Centres for Disease Control and Prevention (CDC) from conducting research on gun violence. They did so because primarily conservative politicians and gun rights advocates argued it would inevitably lead to policy and legislative recommendations for gun control which should be outside the scope of the CDC [44, 45]. Regarding the recommendations that would likely follow this body of research, they were right. Of course, public health recommendations based on the outcome of gun violence research would include greater gun control; it is one of the most well established strategies for curbing gun related violence [46]. Often, however, those arguing for allowing the research has not addressed this; instead, they only continued to promote the abstract and general value of public health research [45, 47, 48]. Organizations such as the National Rifle Association do not explicitly question this but reject its relevance.

When proponents of abstract, value-neutral public health pretend that policy does not ever follow directly from research findings, they limit the impact of their analysis. This pretense also leaves the field open to criticism from those who perceive the direct and obvious conflicts between the values inherent in a public health model and their own political goals. While the case of United States gun control may exemplify this, we can also observe this process more generally. The chaos of many COVID-19 responses, across a variety of jurisdictions, can be partly attributed to a refusal to acknowledge this basic disconnect between epidemiological findings and the sociocultural world to which they had to be applied. As people grasped for some sense of certainty during a pandemic and the Twitter followings of various epidemiologists exploded, those specialist’s insights were rarely effectively acted upon. Why? They seldom offered any engagement with the theories and values upon which this implementation would be based.

## Conclusions

Should we apply a public health perspective to the range of complex issues discussed here? Yes. But this work will be impeded if one presents the analysis as having finally unveiled the true nature of a problem. Instead, we must acknowledge public health analysis as an optional analytical stance that must be applied within a richly elaborated value structure. Rather than acknowledging and integrating the political and theoretical foundations of public health, the field is now often invoked as a rhetorical move, used to construct issues as simple questions of neoliberal scientistic rationalism. Responses to COVID-19 based in sound ‘public health logic’ have provided new opportunities for invasive and sometimes authoritarian erosions of privacy and exacerbated marginalisation [49]. If couching interventions in public health language helps them appear rational and proportional by default, then public health can be used to obscure as much as to illuminate. Everything can be public health, just as everything can be political. Public health is only one essential viewpoint on these chronic and critical problems.

Public health represents a significant breakthrough in understanding human wellbeing, but it is not a god’s eye view that will show us the path out of the mess that humanity has always been in. There is no way out of the systems within which we are embedded, because we are the system. Practitioners should remain confident that surrendering the façade of neutrality will not damage public health, but instead allow it to regain its stature as an integrated scientific, political, and moral force.

## Definitions

*Enlightened rationality*—A way of thinking based on the belief that all real knowledge is arrived at through using logic and that human behaviour should be based on this form of abstract reasoning. It is derived from the principles of the scientific method often attributed to the ‘Enlightenment’ era.

*Individual agency*—The capacity of a person to generate their own desires or intentions, and effectively act upon them.

*Individualism*—The social theory and personal belief that the actions and desires of individual people should be privileged over collective or state control.

*Neoliberal*—A form of governing based on deregulation and reducing state responsibility, expecting that individuals manage their own health and wellbeing.

*Relational autonomy*—An understanding of individual agency and autonomy as always experienced and expressed through relations with people and the environment.

*Scientistic*—The belief that science and the scientific method are the only valid means of generating usable or worthwhile knowledge, and an exaggerated confidence in human’s ability to conduct and learn from scientific experimentation.

*Social justice*—Justice in terms of the distribution of wealth, opportunities, and privileges within a society.

*Solidarity*—Members of a group morally and politically supporting each other due to shared interests and beliefs.

*Structural change*—Changes in the institutions, regulations, economic model, or environment which shape and govern society.
